# Supplementary material for: COZOID: contact zone identifier for visual analysis of protein-protein interactions
Source: BMC Bioinformatics. 2018 Apr 6;19:125. doi: 10.1186/s12859-018-2113-6 (PMC5889581; doi:10.1186/s12859-018-2113-6)
Supplement: Supplementary file 2 — Software build. Executable binary file of the software tool. (ZIP 118,784 kb) [file 12859_2018_2113_MOESM2_ESM.zip › cozoid/java/docs/beansbinding-1.2.1-doc.zip!/org/jdesktop/el/ListELResolver.html]

ListELResolver (Beans Binding)


---


|  |  |  |
| --- | --- | --- |
| **Overview** \| **Package** \| **Class** \| **Use** \| **Tree** \| **Deprecated** \| **Index** \| **Help** | |  |
| **PREV CLASS**   **NEXT CLASS** | **FRAMES**    **NO FRAMES**     **All Classes** |  |
| SUMMARY: NESTED \| FIELD \| CONSTR \| METHOD | DETAIL: FIELD \| CONSTR \| METHOD |  |


---


## org.jdesktop.el Class ListELResolver

```
java.lang.Object
  org.jdesktop.el.ELResolver
      org.jdesktop.el.ListELResolver
```

---

``` public class ListELResolver extends ELResolver ```

Defines property resolution behavior on instances of `List`.

This resolver handles base objects of type `java.util.List`.
It accepts any object as a property and coerces that object into an
integer index into the list. The resulting value is the value in the list
at that index.

This resolver can be constructed in read-only mode, which means that
`isReadOnly` will always return `true` and
`setValue(org.jdesktop.el.ELContext, java.lang.Object, java.lang.Object, java.lang.Object)` will always throw
`PropertyNotWritableException`.

`ELResolver`s are combined together using
`CompositeELResolver`s, to define rich semantics for evaluating
an expression. See the javadocs for `ELResolver` for details.

**Since:**
:   JSP 2.1

**See Also:**: `CompositeELResolver`, `ELResolver`, `List`

---

**Field Summary**

**Fields inherited from class org.jdesktop.el.ELResolver**

`RESOLVABLE_AT_DESIGN_TIME, TYPE`


**Constructor Summary**

`ListELResolver()`
  
          Creates a new read/write `ListELResolver`.

`ListELResolver(boolean isReadOnly)`
  
          Creates a new `ListELResolver` whose read-only status is
determined by the given parameter.


| **Method Summary** | |
| --- | --- |
| `java.lang.Class<?>` | `getCommonPropertyType(ELContext context, java.lang.Object base)`             If the base object is a list, returns the most general type that this resolver accepts for the `property` argument. |
| `java.util.Iterator<java.beans.FeatureDescriptor>` | `getFeatureDescriptors(ELContext context, java.lang.Object base)`             Always returns `null`, since there is no reason to iterate through set set of all integers. |
| `java.lang.Class<?>` | `getType(ELContext context, java.lang.Object base, java.lang.Object property)`             If the base object is a list, returns the most general acceptable type for a value in this list. |
| `java.lang.Object` | `getValue(ELContext context, java.lang.Object base, java.lang.Object property)`             If the base object is a list, returns the value at the given index. |
| `boolean` | `isReadOnly(ELContext context, java.lang.Object base, java.lang.Object property)`             If the base object is a list, returns whether a call to `setValue(org.jdesktop.el.ELContext, java.lang.Object, java.lang.Object, java.lang.Object)` will always fail. |
| `void` | `setValue(ELContext context, java.lang.Object base, java.lang.Object property, java.lang.Object val)`             If the base object is a list, attempts to set the value at the given index with the given value. |

**Methods inherited from class java.lang.Object**

`clone, equals, finalize, getClass, hashCode, notify, notifyAll, toString, wait, wait, wait`

**Constructor Detail**

### ListELResolver

```
public ListELResolver()
```

:   Creates a new read/write `ListELResolver`.

---


### ListELResolver

```
public ListELResolver(boolean isReadOnly)
```

:   Creates a new `ListELResolver` whose read-only status is
    determined by the given parameter.

    **Parameters:**: `isReadOnly` - `true` if this resolver cannot modify lists; `false` otherwise.


**Method Detail**

### getType

```
public java.lang.Class<?> getType(ELContext context,
                                  java.lang.Object base,
                                  java.lang.Object property)
```

:   If the base object is a list, returns the most general acceptable type
    for a value in this list.

    If the base is a `List`, the `propertyResolved`
    property of the `ELContext` object must be set to
    `true` by this resolver, before returning. If this property
    is not `true` after this method is called, the caller
    should ignore the return value.

    Assuming the base is a `List`, this method will always
    return `Object.class`. This is because `List`s
    accept any object as an element.

    :   **Specified by:**: `getType` in class `ELResolver`
    :   **Parameters:**: `context` - The context of this evaluation.: `base` - The list to analyze. Only bases of type `List` are handled by this resolver.: `property` - The index of the element in the list to return the acceptable type for. Will be coerced into an integer, but otherwise ignored by this resolver. **Returns:**: If the `propertyResolved` property of `ELContext` was set to `true`, then the most general acceptable type; otherwise undefined. **Throws:**: `PropertyNotFoundException` - if the given index is out of bounds for this list.: `java.lang.NullPointerException` - if context is `null`: `ELException` - if an exception was thrown while performing the property or variable resolution. The thrown exception must be included as the cause property of this exception, if available.

---


### getValue

```
public java.lang.Object getValue(ELContext context,
                                 java.lang.Object base,
                                 java.lang.Object property)
```

:   If the base object is a list, returns the value at the given index.
    The index is specified by the `property` argument, and
    coerced into an integer. If the coercion could not be performed,
    an `IllegalArgumentException` is thrown. If the index is
    out of bounds, `null` is returned.

    If the base is a `List`, the `propertyResolved`
    property of the `ELContext` object must be set to
    `true` by this resolver, before returning. If this property
    is not `true` after this method is called, the caller
    should ignore the return value.

    :   **Specified by:**: `getValue` in class `ELResolver`
    :   **Parameters:**: `context` - The context of this evaluation.: `base` - The list to be analyzed. Only bases of type `List` are handled by this resolver.: `property` - The index of the value to be returned. Will be coerced into an integer. **Returns:**: If the `propertyResolved` property of `ELContext` was set to `true`, then the value at the given index or `null` if the index was out of bounds. Otherwise, undefined. **Throws:**: `java.lang.IllegalArgumentException` - if the property could not be coerced into an integer.: `java.lang.NullPointerException` - if context is `null`.: `ELException` - if an exception was thrown while performing the property or variable resolution. The thrown exception must be included as the cause property of this exception, if available.

---


### setValue

```
public void setValue(ELContext context,
                     java.lang.Object base,
                     java.lang.Object property,
                     java.lang.Object val)
```

:   If the base object is a list, attempts to set the value at the
    given index with the given value. The index is specified by the
    `property` argument, and coerced into an integer. If the
    coercion could not be performed, an
    `IllegalArgumentException` is thrown. If the index is
    out of bounds, a `PropertyNotFoundException` is thrown.

    If the base is a `List`, the `propertyResolved`
    property of the `ELContext` object must be set to
    `true` by this resolver, before returning. If this property
    is not `true` after this method is called, the caller
    can safely assume no value was set.

    If this resolver was constructed in read-only mode, this method will
    always throw `PropertyNotWritableException`.

    If a `List` was created using
    `Collections.unmodifiableList(java.util.List extends T)`, this method must
    throw `PropertyNotWritableException`. Unfortunately,
    there is no Collections API method to detect this. However, an
    implementation can create a prototype unmodifiable `List`
    and query its runtime type to see if it matches the runtime type of
    the base object as a workaround.

    :   **Specified by:**: `setValue` in class `ELResolver`
    :   **Parameters:**: `context` - The context of this evaluation.: `base` - The list to be modified. Only bases of type `List` are handled by this resolver.: `property` - The index of the value to be set. Will be coerced into an integer.: `val` - The value to be set at the given index. **Throws:**: `java.lang.ClassCastException` - if the class of the specified element prevents it from being added to this list.: `java.lang.NullPointerException` - if context is `null`, or if the value is `null` and this `List` does not support `null` elements.: `java.lang.IllegalArgumentException` - if the property could not be coerced into an integer, or if some aspect of the specified element prevents it from being added to this list.: `PropertyNotWritableException` - if this resolver was constructed in read-only mode, or if the set operation is not supported by the underlying list.: `ELException` - if an exception was thrown while performing the property or variable resolution. The thrown exception must be included as the cause property of this exception, if available.

---


### isReadOnly

```
public boolean isReadOnly(ELContext context,
                          java.lang.Object base,
                          java.lang.Object property)
```

:   If the base object is a list, returns whether a call to
    `setValue(org.jdesktop.el.ELContext, java.lang.Object, java.lang.Object, java.lang.Object)` will always fail.

    If the base is a `List`, the `propertyResolved`
    property of the `ELContext` object must be set to
    `true` by this resolver, before returning. If this property
    is not `true` after this method is called, the caller
    should ignore the return value.

    If this resolver was constructed in read-only mode, this method will
    always return `true`.

    If a `List` was created using
    `Collections.unmodifiableList(java.util.List extends T)`, this method must
    return `true`. Unfortunately, there is no Collections API
    method to detect this. However, an implementation can create a
    prototype unmodifiable `List` and query its runtime type
    to see if it matches the runtime type of the base object as a
    workaround.

    :   **Specified by:**: `isReadOnly` in class `ELResolver`
    :   **Parameters:**: `context` - The context of this evaluation.: `base` - The list to analyze. Only bases of type `List` are handled by this resolver.: `property` - The index of the element in the list to return the acceptable type for. Will be coerced into an integer, but otherwise ignored by this resolver. **Returns:**: If the `propertyResolved` property of `ELContext` was set to `true`, then `true` if calling the `setValue` method will always fail or `false` if it is possible that such a call may succeed; otherwise undefined. **Throws:**: `PropertyNotFoundException` - if the given index is out of bounds for this list.: `java.lang.NullPointerException` - if context is `null`: `ELException` - if an exception was thrown while performing the property or variable resolution. The thrown exception must be included as the cause property of this exception, if available.

---


### getFeatureDescriptors

```
public java.util.Iterator<java.beans.FeatureDescriptor> getFeatureDescriptors(ELContext context,
                                                                              java.lang.Object base)
```

:   Always returns `null`, since there is no reason to
    iterate through set set of all integers.

    The `getCommonPropertyType(org.jdesktop.el.ELContext, java.lang.Object)` method returns sufficient
    information about what properties this resolver accepts.

    :   **Specified by:**: `getFeatureDescriptors` in class `ELResolver`
    :   **Parameters:**: `context` - The context of this evaluation.: `base` - The list. Only bases of type `List` are handled by this resolver. **Returns:**: `null`. **See Also:**: `FeatureDescriptor`

---


### getCommonPropertyType

```
public java.lang.Class<?> getCommonPropertyType(ELContext context,
                                                java.lang.Object base)
```

:   If the base object is a list, returns the most general type that
    this resolver accepts for the `property` argument.
    Otherwise, returns `null`.

    Assuming the base is a `List`, this method will always
    return `Integer.class`. This is because `List`s
    accept integers as their index.

    :   **Specified by:**: `getCommonPropertyType` in class `ELResolver`
    :   **Parameters:**: `context` - The context of this evaluation.: `base` - The list to analyze. Only bases of type `List` are handled by this resolver. **Returns:**: `null` if base is not a `List`; otherwise `Integer.class`.


---


|  |  |  |
| --- | --- | --- |
| **Overview** \| **Package** \| **Class** \| **Use** \| **Tree** \| **Deprecated** \| **Index** \| **Help** | |  |
| **PREV CLASS**   **NEXT CLASS** | **FRAMES**    **NO FRAMES**     **All Classes** |  |
| SUMMARY: NESTED \| FIELD \| CONSTR \| METHOD | DETAIL: FIELD \| CONSTR \| METHOD |  |


---
